# Delayed initiation of breastfeeding in Bukavu, South Kivu, eastern Democratic Republic of the Congo: a cross-sectional study

**DOI:** 10.1186/s13006-018-0150-4

**Published:** 2018-02-13

**Authors:** Richard Mbusa Kambale, Jérémie Bisimwa Buliga, Nancy Francisca Isia, Adolphe Nyakasane Muhimuzi, Oreste Battisti, Bruno Masumbuko Mungo

**Affiliations:** 1 Faculty of Medicine, Catholic University of Bukavu, Bukavu, South-Kivu Province Democratic Republic of the Congo; 2 Department of Pediatrics, Reference Provincial General Hospital of Bukavu, Bukavu, South-Kivu Province Democratic Republic of the Congo; 3 Department of Pediatrics, University of Liège, Liège, Belgium

**Keywords:** Breastfeeding, Initiation, Timely, Delayed, Early, Newborn

## Abstract

**Background:**

Timely initiation of breastfeeding can decrease neonatal mortality. However, about 50% of newborns are not breastfeed within 1 h of birth in the Democratic Republic of Congo. The aim of this study was to identify factors associated with delayed initiation of breastfeeding in an urban and rural area of Bukavu, South Kivu province, Democratic Republic of Congo.

**Methods:**

We interviewed 396 mother-newborn pairs (185 in the urban area and 211 in the rural area) between 20 July and 10 October 2016. We used descriptive statistics to demonstrate the prevalence of early initiation of breastfeeding. Variables that showed association with delayed initiation of breastfeeding in the bivariate models were entered in a multivariable logistic model.

**Results:**

Overall, the rate of early initiation of breastfeeding was 65.9% (69.7% in the rural area, 61.6% in the rural area). Two hundred and seventy-four (62.9%) mothers (159 in rural area and 115 in urban area) were counselled on early initiation of breastfeeding during prenatal care. Most mothers, 65.2% received counselling by a health professional. On multivariable regression analyses after adjusting for other variables in the model, unmarried mothers [Odds Ratio (OR): 1.5 (95% Confidence Interval (CI): 1.13, 1.95)], cesarean delivery [OR: 2.24 (95% CI: 1.74, 2.88)], no counselling on timely initiation of breastfeeding [OR: 1.71 (95% CI: 1.29, 2.20)] and counselling by a non-health professional [OR: 1.84 (95% CI: 1.08, 3.12)] were associated with delayed initiation of breastfeeding.

**Conclusion:**

Systemic changes are needed for women having caesarean births to experience skin-to-skin and early initiation. In addition, information, education and communication on the importance of timely initiation of breastfeeding must be supported to improve maternal and infant wellbeing.

## Background

Neonatal mortality rate represents a large proportion of under-5 mortality, despite a significant reduction in child mortality from 12.7 million in 1990 to 5.9 million in 2015 [1]. Globally, neonatal mortality represented approximately 45% of under-5 deaths in 2015 [2]. Reducing neonatal mortality remains, therefore, the main priority in infant survival. Breastfeeding is a unique, valuable feeding practice in infancy that is associated with lower neonatal mortality and which alleviates inequities in child mortality and prevents morbidities such as diarrhoea, pneumonia, neonatal sepsis and may reduce obesity and diabetes later in life [3–6]. The global breastfeeding recommendations are to place all newborns in skin-to-skin contact with their mothers immediately after birth, to support timely initiation of breastfeeding (defined as the initiation of breastfeeding within 1 h after birth) and to exclusively breastfeed the child until 6 months of age. Early initiation of breastfeeding stimulates breast milk production, produces antibody protection for the newborn and reduces postpartum maternal haemorrhage, and its practice determines the successful establishment and longer duration of breastfeeding [6, 7].

Despite the known health benefits of early initiation of breastfeeding, a considerable proportion of newborns are not breastfed within 1 h after birth in accordance with the WHO recommendation in many regions. Worldwide, the prevalence of early initiation of breastfeeding is 44%. A systemic assessment at national level in the countries of south Asia revealed that the initiation of breastfeeding within 1 h is achieved in 39% babies. In Africa, the lowest early initiation of breastfeeding rates are found in West and Central Africa (40%) and Middle East and North Africa (44%), while the highest rate is found in Eastern and Southern Africa (59%) [8]. In the Democratic Republic of Congo (DRC), the rate of early initiation of breastfeeding has increased slightly from 48.1% in 2007 [9] to 51.9% in 2015 [8, 10].

Exclusive breastfeeding is not only the easiest, cost effective and most successful intervention; it also tops the table of life-saving interventions for the health of the newborn [11–13]. Several studies have reported that breastfeeding education is an effective way to increase both the early initiation of breastfeeding and the duration of breastfeeding [14–18]. Little is known about factors predisposing to delayed initiation of breastfeeding in the DRC. Moreover, in the DRC, the 2014 Demographic and Health Survey provided an overall description of the practices observed, but these national data hide regional, provincial or cultural disparities. The objectives of this study were (i) to evaluate the prevalence of early initiation of breastfeeding, (ii) to investigate the most important determinants of delayed initiation of breastfeeding in South Kivu Province and (iii) to assess the impact of breastfeeding education on early initiation of breastfeeding.

## Method

### Study area

This cross-sectional study was carried out in two maternity wards: the maternity wards of Reference Provincial General Hospital of Bukavu (RPGHB), an urban maternity ward, and the maternity ward of Miti-Murhesa General Hospital (MGH), a rural one. The maternity wards of RPGHB records about 3000 admissions and 1500 to 2000 births per year. The RPGHB is one of the main healthcare facilities in Bukavu, a city of more than 700,000 inhabitants in South Kivu Province in eastern DRC. It organizes tertiary care services. The MGH is the reference structure of the rural health zone of Miti-Murhesa which has 18 health centers and a general reference hospital. It records about 1200 to 1500 deliveries per year.

### Sample size

The study population was constituted by the mother-neonate pairs monitored in the study maternity wards from 20 July to 10 October 2016. The size of our sample was calculated by the formula: $\mathrm{n}=\frac{{\mathrm{t}}^2\bullet \mathrm{p}\bullet \left(1-\mathrm{p}\right)\ }{{\mathrm{m}}^2} = \frac{(1.96)^2\bullet 0.519\bullet 0.481\ }{(0.05)^2} \approx 384\ \mathrm{individuals}$. [n: sample size; t: 95% confidence level (typical value 1.96); p: probability of early initiation of breastfeeding (51.9% according to Demographic and Health Surveys DRC 2014); m: margin of error (5%)]. According to the Demographic and Health Survey-DRC 2014, the population concerned by the early initiation of breastfeeding is unequally represented; 47.6% of early initiation of breastfeeding are in urban areas and 52.4% in rural areas. Taking this into account, the number of individuals to be interviewed was determined as follows: RPGHB (urban) =183 individuals; MGH (rural) = 201 individuals.

### Inclusion and exclusion criteria

The study included mother-neonates pairs with the following characteristics: (i) giving a live birth in the maternity wards of RPGHB or MGH during study period, (ii) giving birth to a single neonate (iii), having a workable medical record, (iv) informed consent of the mother obtained. The study population was restricted to neonates and mothers considered able to initiate breastfeeding and excluded those with multiple births, pairs with missing data on initiation of breastfeeding, mothers declining to participate in the survey and mothers with certain maternal and perinatal complications, including congenital malformation, neonatal near-miss cases at gestational age < 33 weeks, birth weight < 1750 g or Apgar score at 5 min < 7, women with severe maternal outcomes defined as the presence of any of the following conditions: eclampsia, blood transfusion, hysterectomy, admission to intensive care units, deliveries with general anaesthesia and maternal death.

### Data collection

The data were collected using a survey form. A pilot test of the questionnaire was performed. The dependent variable was the initiation of breastfeeding. This continuous variable was subdivided into 2 groups: (i) early initiation of breastfeeding, if breastfeeding initiation had occurred within 1 h of birth; and (ii) delayed initiation of breastfeeding, if breastfeeding initiation had occurred more than 1 h after birth. The independent variables studied were maternal age, parity, interpregnancy interval, marital status, mother’s schooling status, socioeconomic status, parent occupation, number of prenatal care visits, breastfeeding education during prenatal care visits, breastfeeding educator, gestational age, mode of delivery, newborn gender, birth weight, number of breastfeeds in the first 24 h / 48 h / 72 h and the sucking reflex.

The breastfeeding education guideline of the study area was: (i) to place newborn in skin-to-skin contact with his mother immediately after birth, (ii) to initiate breastfeeding within 1 h after birth and (iii) to exclusively breastfeed the child until 6 months of age. Breastfeeding education was described as “good” if the mother knew at least 2 of these 3 items, and “bad” if the mother knew only one item or none. In the study, we used the term “Health professional” to designate midwives, nurses or physician. We used the concept “Non-health professional” for other persons who do not belong to this category. Preterm newborn was defined as an infant born before 37 weeks of gestational age and a newborn at full-term infant as an infant born between 37 and 42 weeks of gestational age. The socioeconomic status was determined on the basis of a household wealth score as proposed by Bangirana et al. [19] and Filmer et al. [20] on the basis of the household’s material assets and on the basis of several characteristics of the house such as materials used for walls and for the roof. The household wealth score was obtained by adding up the points assigned to the above items.

### Statistical analysis

The data were checked for completeness, inconsistencies, entered, coded, cleaned and analyzed using SPSS for Windows Version 20 (SPSS Inc. Version 20.0, Chicago, Illinois). Descriptive statistics was computed to determine the prevalence of early initiation of breastfeeding. Proportions were compared by using either the χ^2^ or the Fisher exact test. The nonparametric Kruskall-Wallis test was used for the comparison of quantitative variables. Incidence rate ratios (IRR) and their 95% confidence intervals (95% CI) were computed by using the group in which the incidence was the lowest as reference. First a bivariate logistic regression was performed. Variables that showed significant association with delayed initiation of breastfeeding in the bivariate models were entered in a multivariable logistic model. To identify independent predictors of delayed initiation of breastfeeding, a multivariable logistic regression model with delayed initiation of breastfeeding as dependent variable was constructed and a *p* <  0.05 was considered statistically significant.

## Results

We interviewed 396 mother-newborn pairs (185 at RPGHB and 211 at MGH). The characteristics of all mothers and newborns are summarized in Table 1. Overall, the rate of early initiation of breastfeeding was 65.9% (69.7% in the rural area, 61.6% in the urban area); although the evidence for a difference was weak (*p* = 0.09).

**Table 1:** Characteristics of study sample (*n* = 396)

Variables (n = 396)	Rural n (%)		Urban n (%)		Total	
Maternity ward	211 (53.3)		185 (46.7)		396 (100)	
Mother’s age [median (range)]		25 (17 – 46)		28 (17 – 43)	27 (17 – 46)	
< 18	7 (3.3)		5 (2.7)		12 (3.0)	
18 – 35	178 (84.4)		154 (83.2)		332 (83.8)	
≥ 35	26 (12.3)		26 (14.1)		52 (13.2)	
Inter pregnancy interval		26 (12 – 97)		27 (11 – 108)		26 (11 – 108)
< 24 months	50 (29.9)		50 (36.2)		100 (32.8)	
≥ 24 months	117 (70.1)		88 (63.8)		205 (67.2)	
Marital status						
Married	160 (75.8)		103 (55.7)		263 (66.4)	
Unmarried	51 (24.2)		82 (44.3)		133 (33.6)	
Mother’s schooling status						
Unschooled	99 (46.9)		12 (6.5)		111 (28.0)	
Primary school	69 (32.8)		32 (17.3)		101 (25.5)	
Secondary school	37 (17.5)		90 (48.6)		127 (32.0)	
University studies	2 (0.9)		46 (24.9)		48 (12.1)	
Technical school	4 (1.9)		5 (2.7)		9 (2.4)	
Socioeconomic status						
Low	204 (96.7)		51 (27.6)		255 (64.4)	
Medium	4 (1.9)		71 (38.4)		75 (18.9)	
Good	3 (1.4)		63 (34.1)		66 (16.7)	
Parity (Mean ± SD)		4 ± 3		3 ± 2		4 ± 2
Primipara	40 (19.0)		49 (26.5)		89 (22.5)	
Multipara	171 (81.0)		136 (73.5)		307 (77.5)	
Number of PCV (Mean ± SD)		3 ± 1		3 ± 1		3 ± 1
< 3	78 (37.0)		48 (25.9)		126 (31.8)	
≥ 3	133 (63.0)		137 (74.1)		270 (68.2)	
HIV Serology						
Positive	9 (4.3)		2 (1.0)		11 (2.8)	
Negative	168 (79.6)		183 (99.0)		351 (88.6)	
Unknown	34 (16.1)		0		34 (9.2)	
Mode of delivery						
Vaginal delivery	146 (69.2)		157 (84.9)		303 (76.5)	
Cesarean delivery	65 (30.8)		28 (15.1)		93 (23.5)	
Birth weight [median (range)] (kg)		3.1 (1.8 – 4.8)		3.2 (1.9 – 5.2)		3.2 (1.2 – 5.2)
< 2.5	20 (9.5)		15 (8.1)		35 (8.8)	
2.5 – 4.0	183 (16.7)		160 (86.5)		343 (86.6)	
≥ 4.0	8 (3.8)		10 (5.4)		18 (4.6)	
Gestational age						
At term	199 (94.8)		171 (92.4)		370 (93.4)	
Preterm	11 (5.2)		15 (7.6)		26 (6.6)	
Gender						
Male	97 (46.0)		95 (51.4)		192 (48.5)	
Female	114 (54.0)		90 (48.6)		204 (51.5)	

*PCV* prenatal care visits, *SD* standard deviation, *HIV* human immunodepression virus

**Fig. 1:**
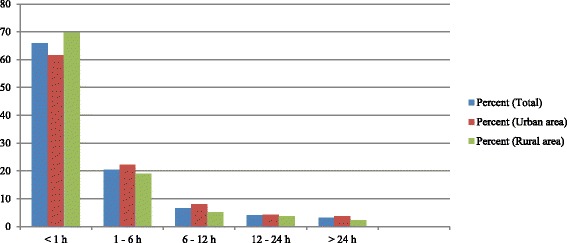
Distribution of breastfeeding initiation of the 396 mother-newborn pairs interviewed

**Fig. 2:**
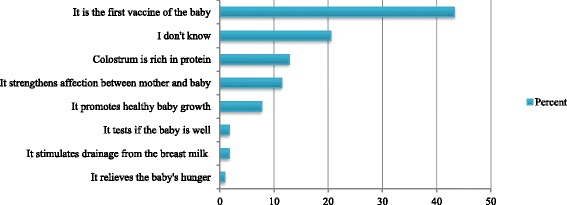
Knowledge status of the 396 mothers interviewed about advantages of early initiation of breastfeeding and colostrum

Figure 1 shows the distribution of breastfeeding initiation during the first 24 h of life. For newborns who were breastfed early, the median time to breastfeeding after birth was 15 (IQR 5 - 30) minutes. Two hundred and seventy-four (62.9%) mothers (159 in rural area and 115 in urban area) were counselled on early initiation of breastfeeding during prenatal visits. In 258 (65.2%) mothers (154 rural and 104 urban), this was done by a health professional as shown in Table 2. Two hundred and forty-three (69.2%) mothers recognized the importance of early initiation of breastfeeding (immune role, stimulates drainage from the breast milk, nutritional benefits, strengthening the mother-child psycho-affective link) as shown in Fig. 2.

**Table 2:** Breastfeeding education and breastfeeding educator of 396 mother-newborn pairs interviewed

Variables n = 396	Rural n (%)	Urban n (%)
Quality of breastfeeding education^a^		
None	81 (50.0)	54 (23.0)
Bad	31 (19.1)	55 (23.5)
Good	50 (30.9)	125 (53.5)
Time to first breastfeed		
Median (IQR)	50 min (15 – 120)	57 min (15 – 180)
Median (Range)	50 min (0 – 4320)	57 min (0 – 4320)
Early initiation of breastfeeding		
Yes	147 (69.7)	114 (61.6)
No	64 (30.3)	71 (38.4)
Breastfeeding education before this pregnancy		
Yes	153 (72.5)	110 (59.5)
No	58 (27.5)	75 (40.5)
Breastfeeding education during this pregnancy		
Yes	159 (75.4)	115 (62.2)
No	52 (24.6)	70 (37.8)
Breastfeeding education before and during this pregnancy		
Yes	149 (70.6)	100 (54.1)
No	62 (29.4)	85 (45.9)
Breastfeeding counsellor (*n* = 274)		
Family	4 (2.5)	3 (2.6)
Neighbour	1 (0.6)	1 (0.9)
Personal documentation	0	7 (6.1)
Health professional (midwives, nurses or physician)	154 (96.9)	104 (90.4)

*IQR* interquartile range

^a^ The breastfeeding education guideline of the study area was: (i) to place newborn in skin-to-skin contact with his mother immediately after birth, (ii) to initiate breastfeeding within 1 h after birth and (iii) to exclusively breastfeed the child until 6 months of age. Breastfeeding education was described as “good” if the mother knew at least 2 of these 3 items, and “bad” if the mother knew only one item or none

During the early initiation of breastfeeding, the sucking reflex was good in 355 (89.6%) newborns. Overall, the average number of feedings in the first 24 h (6 feeds), first 48 h (12 feeds) and first 72 h (20 feeds) were slightly lower than recommended (one feeding on average every 3-4 h). But on the 3rd postpartum day, the feeding frequency was higher in the urban group than in the rural one (*p* <  0.000).

Table 3 compares the impact of breastfeeding education during prenatal care on early initiation of breastfeeding in both groups. Mothers who were not counselled / advised on early initiation of breastfeeding were 1.5 times as likely to delay breastfeeding initiation than those who were counselled [OR: 1.53 (95% CI: 1.17, 2.00)]. Similarly, mothers who were counselled / advised on early initiation of breastfeeding by a non-health professional were 1.8 times as likely to practice delayed initiation compared to those who were counselled by health professional [OR:1.84 (95% CI:1.08, 3.12].

**Table 3:** Early initiation of breastfeeding according to moment of sensitization, breastfeeding educator and knowledge status of the 396 mothers interviewed

Variables	Delayed initiation of breastfeeding n (%)	Early initiation of breastfeeding n (%)	IRR (95%CI)	*p*-value
Breastfeeding education before this pregnancy				
No	59 (44.4)	74 (55.6)	1.53 (1.17, 2.00)	0.002
Yes	76 (28.9)	187 (71.1)	1	
Breastfeeding education during this pregnancy				
No	58 (47.5)	64 (52.5)	1.69 (1.29, 2.20)	< 0.000
Yes	77 (28.1)	197 (71.9)	1	
Breastfeeding education before and during this pregnancy				
No	64 (43.5)	83 (56.5)	1.52 (1.16, 1.91)	0.002
Yes	71 (28.5)	178 (71.5)	1	
Breastfeeding educator				
Other	8 (50.0)	8 (50.0)	1.84 (1.08, 3.12)	0.049
Health professional (midwives, nurses or doctors)	70 (27.1)	188 (72.9)	1	
Better knowledge of the importance of early initiation of breastfeeding				
Non	30 (34.9)	56 (65.1)	1.18 (0.81 – 1.71)	0.39
Yes	48 (29.6)	114 (70.4)	1	

*IRR* Incidence rate ratios

On multivariable regression analyses after adjusting for other variable in the model, marital status, mode of delivery, counselling about early initiation of breastfeeding and professional status of the counsellor were independent predictors of delayed initiation of breastfeeding. Unmarried mothers were 1.5 times as likely to practice delayed initiation compared to their married counterparts [AOR: 1.5 (95% CI: 1.13, 1.95)]. Mothers with caesarean delivery were 2.2 times as likely to practice delayed initiation compared to those with vaginal delivery [AOR: 2.24 (95% CI: 1.74, 2.88)]. Similarly, mothers who were not counselled on early initiation of breastfeeding and those who were counselled on early initiation of breastfeeding by a non-health professional were, respectively 1.7 times [AOR: 1.71 (95% CI: 1.29, 2.20)]; and 1.8 times [AOR: 1.84 (95% CI: 1.08, 3.12)] to practice delayed initiation as shown in Table 4.

**Table 4:** Multivariate analysis of different risk factors of delayed initiation of breastfeeding for 396 mothers

Variables	Delayed initiation of breastfeeding n (%)	Early initiation of breastfeeding n (%)	AOR (95%CI)	p-value
Marital status				
Unmarried	58 (43.6)	75 (56.4)	1.49 (1.13, 1.95)	0.004
Married	77 (29.3)	186 (70.7)	1	
Mode of delivery				
Cesarean delivery	55 (59.1)	38 (40.9)	2.24 (1.74, 2.88)	< 0.000
Vaginal delivery	80 (26.4)	223 (73.6)	1	
Counselling about early initiation of breastfeeding				
No	59 (44.4)	74 (55.6)	1.71 (1.29, 2.20)	< 0.000
Yes	76 (28.9)	187 (71.1)	1	
Who counselled about early initiation of breastfeeding?				
Non- Health professional	8 (50.0)	8 (50.0)	1.84 (1.08, 3.12)	0.049
Health professional	70 (27.1)	188 (72.9)	1	

*AOR* adjusted odds ratio, *CI* confidence Interval

## Discussion

The aim of this study was to evaluate the prevalence of early initiation of breastfeeding including factors associated with delayed initiation of breastfeeding among mothers at RPGHB and MGH, in Bukavu, South Kivu province, eastern of DRC. We investigated breastfeeding initiation among women and their singleton neonates without maternal and perinatal severe adverse outcomes. The prevalence of early initiation of breastfeeding was 65.9%. We found that unmarried mothers, cesarean delivery, no counselling on early initiation of breastfeeding and the counselling by a non-health professional during prenatal care visits were associated with delayed initiation. Factors at geographical, socioeconomic and individual, such as residence, education, household wealth, family size, occupation, interpregnancy interval, mother’s age and newborn’s gender and term were not found to be associated with delayed initiation of breastfeeding.

The prevalence of early initiation of breastfeeding in this study is high compared to that reported throughout the DRC (national) (52%) and in West and Central Africa (40%). In Central Africa, the prevalence of early initiation of breastfeeding is lower in DRC than in Rwanda (71%) and Burundi (74%) [8]. In some developing countries, the prevalence of early initiation of breastfeeding was also documented as in Ethiopia (69%), Ghana (41%), Sudan (54.2%), Zambia (70%), Jordan (49.5%), North Jordan (86.6%), Nepal (72.2%), Bolivia (74%) [21–27]. The prevalence of early initiation of breastfeeding ranges from 14% to 95%, with an average of 64% in 128 countries, and one-half of these countries have a prevalence of less than 50% [8].

We found that cesarean delivery was one of the factors contributing to the delayed initiation of breastfeeding. Other authors have also found this negative association between cesarean delivery and early initiation of breastfeeding [28–30]. In a systematic review of the factors and obstacles to breastfeeding initiation within 1 h of birth in South Asia (Afghanistan, Bangladesh, Bhutan, India, Maldives, Nepal, Pakistan, Sri Lanka and India), the authors identified cesarean delivery as major obstacle to early initiation of breastfeeding [31]. Authors of another recent systematic review and meta-analysis of world literature reported that caesarean delivery was significantly negatively associated with early initiation of breastfeeding [32]. They suggested that maternal and fetal indications for caesarean delivery and postoperative care disrupt bonding and mother-newborn interaction and delayed initiation of breastfeeding. They also found greater risk of delayed initiation in elective, pre-labour caesarean delivery and suggested a possible relationship between maternal preference for caesarean delivery and the decision not to breastfeed [32].

To circumvent the obstacle of the caesarean delivery in the early initiation of breastfeeding, five strategies have been developed to increase early initiation of breastfeeding post-caesarean delivery, including adoption of supportive hospital policies, training of medical staff to support breastfeeding post caesarean delivery, removal of physical barriers, education about caesarean delivery and breastfeeding, and reduction of caesarean deliveries that are not medically indicated [33]. Considering the increasing rates of caesarean delivery globally, it is crucial to encourage and support early initiation of breastfeeding in all women regardless of the mode of delivery and to inform all prospective mothers and health staff of the negative effects of caesarean delivery on breastfeeding and the wellbeing of the newborn.

In this study, the marital status was the only maternal socio-demographic characteristic associated with delayed initiation of breastfeeding, particularly unmarried mothers. Few other studies have found this correlation. Seranath et al. reported a high rate of delayed initiation of breastfeeding in newborns of mothers aged 15-19 years [34]. Other maternal socio-demographic characteristic associated with delayed initiation of breastfeeding reported in systematic review conducted in Asia, Africa and South America between 1999 and 2013 were low family income, maternal age less than 25 years, low maternal education and no prenatal guidance on breastfeeding [35].

Breastfeeding education during prenatal care plays an important role in early initiation of breastfeeding. About 30% of the mothers in our sample were unaware of the benefits of colostrum and early initiation of breastfeeding. This emphasizes the need of breastfeeding counselling, especially during prenatal visits where mothers are advised by health professionals. Breastfeeding counselling, especially when provided by health professionals, is a less costly and effective way to break down sociocultural barriers to breastfeeding. Several obstacles have been described. Lack of availability of information for correct knowledge and misperception on breastfeeding was reported as a barrier. Lack of knowledge on the importance of early initiation and the perception that water must be given to the newborn because breast milk alone will not sustain the baby were observed in Bangladesh [36]. Negative perception of colostrum and the use of prelacteal feeds are common barriers, shown in other studies. In Pakistan women reported discarding colostrum, withholding breastfeeding and replacing with prelacteal feeding which is typically administered via a finger of an elderly person and perceived to clean the stomach and strengthen the newborn [37]. Another study described the perception that colostrum may harm or even kill the newborn because it is dirty and stored for 9 months in the breast [38]. Likewise, in a rural area of India mothers perceive that the first milk is harmful to the baby [39]. Mothers in urban India who accept giving colostrum are more likely to initiate breastfeeding within 1 h of the birth [40].

Formal breastfeeding education is that which is provided over and above the breastfeeding information given as part of standard antenatal care, and which may include individual or group education sessions led by peer counsellors or health professionals, homes visits, lactation consultation, distribution of printed/written materials, video demonstrations and inclusion of prospective fathers in learning activities. The antenatal period affords an opportunity for providing pregnant women and their partners and families with information about the benefits of breastfeeding at a time when many decisions about infant feeding are being contemplated. Systematic reviews of the available evidence suggest that breastfeeding education is effective in increasing both the rate of breastfeeding initiation and breastfeeding duration [14–18].

## Conclusion

Our findings suggest that unmarried mothers, cesarean delivery, no counselling on early initiation of breastfeeding and the counselling by a non-health professional during prenatal care visits were negatively associated with the rate of early initiation of breastfeeding. During group prenatal care visits, special emphasis should be placed on the early initiation of breastfeeding importance and the colostrum role. Prenatal support, hospital management and subsequent pediatric and maternal visits are all-important components of early initiation of breastfeeding promotion. Systemic changes are needed for women having caesarean births to experience skin-to-skin and early initiation.

## Abbreviations

AOR: Adjusted odds ratio
CI: Confidence interval
DRC: Democratic Republic of Congo
HIV: Human immunodepression virus
IRR: Incidence rate ratios
MGH: Miti-Murhesa General Hospital
RPGHB: Reference Provincial General Hospital of Bukavu
SD: Standard deviation
